# Load-Velocity Relationship in Bench Press and Effects of a Strength-Training Program in Wheelchair Basketball Players: A Team Study

**DOI:** 10.3390/ijerph182111161

**Published:** 2021-10-24

**Authors:** Ander Romarate, Aitor Iturricastillo, Fabio Y. Nakamura, Irineu Loturco, Josune Rodriguez-Negro, Cristina Granados, Javier Yanci

**Affiliations:** 1 Department of Physical Education and Sport, Faculty of Education and Sport, University of the Basque Country, UPV/EHU, 01007 Vitoria-Gasteiz, Spain; aromarate002@ikasle.ehu.eus (A.R.); josune.rodriguez.negro@gmail.com (J.R.-N.); cristina.granados@ehu.eus (C.G.); javier.yanci@ehu.es (J.Y.); 2 Department of Medicine and Aging Sciences, “G. d’Annunzio” University of Chieti-Pescara, 66100 Chieti, Italy; fabioy_nakamura@yahoo.com.br; 3 The College of Healthcare Sciences, James Cook University, Brisbane City, QLD 4000, Australia; 4 Nucleus of High Performance in Sport, São Paulo 04023-062, Brazil; irineu.loturco@terra.com.br

**Keywords:** mean propulsive velocity, disability, resistance, training, 1RM

## Abstract

Performance in wheelchair basketball is determined by capabilities, such as strength and power. The study has two aims: first, to analyze the association between speed and acceleration variables (collected in the bench press (BP) exercise) and the distinct percentages of one-repetition maximum (1RM); second, to analyze the effect of a strength training protocol on wheelchair basketball (WB) players according to their functional impairments. Ten Spanish male WB players volunteered to participate in the study. The players did a pretest and posttest (1RM in bench press) with 6-week muscle strength intervention program. The results showed a high association between the %1RM and the mean propulsive velocity (MPV) and the maximum velocity (V_max_), both in the total of the participants, and in each separate group of athletes. After implementing the strength training program, both the players of the IWBF (International Wheelchair Basketball Federation) < 2.5 group and those of IWBF > 2.5 group improved their 1RM (*p* < 0.01, ES = 0.20 to 0.23). However, the program produced positive effects at submaximal intensities in the MPV reached with 30, 40, 70, and 80 kg and in time to maximum velocity (TV_max_) with 30, 40, and 70 kg (ES = −3.24 to 1.32) only in players with greater functional impairments. The high association between %1RM and MPV and V_max_ can allow for determination the %1RM of the WB players in the BP using the MPV and the V_max_. The training program was effective in improving 1RM in both groups, while improvements in submaximal values only occurred in the IWBF < 2.5 group.

## 1. Introduction

Strength and power capacities are amongst the most relevant physical fitness components in many individual [1,2] and team sports [3,4]. Specifically in wheelchair basketball (WB), some previous studies observed that muscular strength is especially relevant for competitive performance [5,6], due to its influence on crucial actions, such as propulsion, acceleration, deceleration, or changes of direction [5]. For this reason, several studies have analyzed the neuromuscular performance of WB players through different field tests, such as the handgrip, the medicine ball throw, the maximal pass [5,7,8], or through non-specific tests, such as bench press (BP), where the load corresponding to the one-repetition maximum (1RM) that an athlete is able to perform can be reliably and accurately determined [6,9,10].

Although several studies have shown that the 1RM test is valid and reliable to determine the muscular strength of athletes [11,12], its time-consuming characteristics make its use more complicated in large groups of individuals (i.e., team sports) [13,14]. In addition, as this measurement is performed under maximum loading conditions, the risk of injury could be high [14,15]. To know if it is possible to predict the percentage of 1RM (%1RM) by simply calculating the velocity developed at submaximal intensities [16], several studies have examined the relationship between the percentages of %1RM and the corresponding mean propulsive velocity (MPV) in able-bodied athletes [12,17]. Iturricastillo et al. [10] also analyzed this relationship in WB players, where a nearly perfect and inverse relationship has been observed between the %1RM and MPV for the BP exercise (i.e., free execution mode). Moreover, these authors determined that it could be interesting to design specific training programs at an MPV of 0.90 to 1.09 m·s^−1^ due to the subjects in the study obtained the maximal power outputs (i.e., Mean Power, Mean Propulsive Power, and Peak Power) in this range of values. On the other hand, a correlation has been observed between the 1RM value and the ability to perform repeated efforts under fatigue conditions [18]. However, to the best of our knowledge no study analyzed the relationship between %1RM and MPV differentiating the high and low point class players.

Different investigations have highlighted the need to improve strength in wheelchair players due to its relevance to WB performance [5,6,19]. Accordingly, two recent studies have analyzed the effect of a competitive season on WB players’ performance showing that the changes observed in the WB strength capacity or physical fitness were mostly trivial or small [20,21]. These authors suggest that it would be interesting to study the effects of implementing specific programs to improve physical performance in WB. Turbanski et al. [6] reported the effects produced by a specific resistance training program in WB and wheelchair rugby players, who presented significant increases in strength and speed qualities after systematically performing BP exercise during 8 weeks. In other Paralympic modalities, such as ice hockey or swimming, it has been observed that strength training is able to produce improvements in maximum strength [22,23], as well as in other neuromuscular abilities, such as the sprint ability [9,22,24]. Nevertheless, WB coaches and physical trainers do not have enough evidence yet to make the decision to introduce (or not) resistance training programs in their player’s routines, as well as how to do it effectively.

Bearing in mind that, in WB, players are classified according to their functional impairments [25], it may be necessary to consider also this aspect in scientific studies. A previous study [26] stated that physical abilities of players may vary, depending on their functional impairments. Specifically with regard to the maximum strength, it has been observed that athletes with greater degrees of impairments have a reduced capacity to apply force [27]. In the same line, specifically in WB players, several studies have concluded that there are differences in the capacity to produce force according to the functional impairment [28,29], being the players of the lower categories the ones with the lowest strength levels. This impaired capacity may be due to the fact that the players with greater impairments have lower muscle mass and activation levels [27], factors that negatively affect strength ability [30]. Although it has been stated that the capacity to generate force may be conditioned by the player’s impairments [31], it is currently unknown if the response to specific strength-power programs may be influenced by the type or degree of such disability. Knowing the effects produced by training programs according to the functional limitations can be important, since WB teams are composed of players with different profiles, and it would be desirable that all players improve their physical performance within individual possibilities.

Therefore, the objectives of this study were: (1) investigate the relationships between different mechanical variables (MPV, V_max_, and TV_max_) and distinct %1RM in BP exercise in WB players, and (2) analyze the effects of a specific strength training protocol in WB players attending the physical impairment.

## 2. Materials and Methods

### 2.1. Participants

Ten Spanish First Division male WB players (27.9 ± 10.3 years, 60.3 ± 12.2 kg) volunteered to participate in the study. In order to define the disability of each player, the International Wheelchair Basketball Federation (IWBF) designed a classification system based on the physical ability of the player to execute the specific movements of basketball: pushing the wheelchair, dribbling, shooting, passing, and catching. In this sense, the classification of players is done to describe different variables, such as volume of action (the limit to which a player can voluntarily move in the vertical plane, frontal plane and lateral plane), sitting position, and pelvic stability [32]. Thus, players are grouped into categories (functional classes) from 1.0 (being the player with the least physical functionality) to 4.5 (being the player with the highest physical functionality). This classification constitutes the “game points” of the players, and, at any time during a match, the five players on court must not exceed a total of 14 game points. In the present study, participants were divided into two groups, according to their functional sports classification (IWBF Player Classification Commission, 2014): IWBF < 2.5 (Total players from class 1 to 2.5 = 4; class 1, *n* = 2, class 1.5, *n* = 1, class 2.5, *n* = 1) and IWBF > 2.5 (Total players from class 3 to 4.5, *n* = 6; class 3, *n* = 1, class 3.5, *n* = 1, class 4, *n* = 2 and class 4.5, *n* = 2). This study was approved by the Ethics Committee of the University of the Basque Country (cod. CEISH—M10_2020_244), and all participants provided written informed consent as outlined in the Declaration of Helsinki (2013).

### 2.2. Procedures

Participants performed a 6-week muscle strength intervention program during the preseason. One week before (pretest) and one week after (posttest) the intervention program, all participants performed a test to determine the 1RM in BP in a free execution mode. Nevertheless, 4 standardized 1RM test familiarization sessions were performed to refine the execution technique.

### 2.3. Measures

#### 2.3.1. Measurement of Muscle Strength

To determine maximum dynamic strength, the 1RM protocol in BP described previously by Sanchez-Medina et al. [33] was performed. Participants were instructed to lower the bar (eccentric phase) in a controlled manner until reaching their chest, perform a 1.5 s stop to avoid the use of the muscle stretch-shortening cycle, and perform the concentric phase at the maximum possible speed. The initial load for all athletes was 20 kg, and progressive increases of 10 kg were made until reaching an MPV lower than 0.5 m·s^−1^. From this speed on, load increases were lower (5–1 kg) in order to determine the 1RM with greater precision [33]. Three repetitions were performed with each light load (MPV > 1.0 m.s^−1^), two with each medium load (0.65 m.s^−1^ < MPV < 1.0 m.s^−1^), and only one with each heavy load (MPV < 0.65 m.s^−1^) [33]. Players made breaks of 3 min between sets in medium and light loads and 6 min between high loads. For analysis purposes, the best repetition performed with each load was used. The highest load (1RM_Load_) that each subject was able to lift once with a total extension of the elbows was considered as 1RM. All force variables were measured with a linear encoder (T-Force System, Ergotech, Murcia, Spain) was used for the measurement of the MPV, the maximum velocity (V_max_), and time to maximum velocity (TV_max_), and the data were processed by the device’s own software [6,34] during the concentric phase of the exercise, both in the 1RM and in the rest of submaximal loads.

#### 2.3.2. Training Protocol

Participants performed a specific training protocol aimed at improving muscle strength, adapted from a study conducted by González-Badillo et al. [35]. The training protocol lasted 6 weeks (three sessions per week), and the exercise used was the BP (Table 1). The participants had 2 min of recovery between sets, and the %1RM used in each session was calculated individually for each athlete using the 1RM obtained in the pretest. Athletes were instructed to perform all the repetitions at the maximum speed possible in their concentric phase, and the eccentric phase of each repetition was performed in a controlled manner. The warm-up before the sessions consisted of 5 min of joint mobility exercises of the upper extremities and in lifting 2 × 10 repetitions (reps) with the 20 kg bar. During the training protocol, the athletes continued performing the usual pre-season training established by the technical staff, which did not include specific content of physical conditioning.

#### 2.3.3. Data Analysis

The results are presented as mean ± standard deviation (SD). Data normal distribution and homogeneity of variances was showed according to the Shapiro-Wilk and Levene tests, respectively. The associations between the MPV and the V_max_ with the %1RM were calculated using the Pearson correlation coefficient (r). For the interpretation of the magnitudes of the correlations, the following scale was used: <0.1, trivial; 0.1–0.29, small; 0.3–0.49, moderate; 0.5–0.69, large; 0.7–0.9, very large; >0.9, nearly perfect [36]. The confidence limits (±CL, 90%) were also calculated, as well as the probabilities that the associations were true [36]. The linear regression formula between the %1RM, the MPV and the V_max_ were calculated. To examine the existence of differences between the IWBF < 2.5 and the IWBF >2.5 groups in the analyzed variables in the pretest, a Mann–Whitney U was used. To determine the differences between the results obtained between the pretest and the posttest, a paired *t*-test of related samples was used for all the players and a Wilcoxon test for each of the groups. The mean differences were calculated in percentage (Δ%) = [(mean posttest—mean pretest) × 100/mean pretest]. To quantify the differences of means for practical purposes, the effect size (ES) [37] was calculated. The scale for the interpretation of the ES was: <0.2, trivial; 0.2 to 0.5, small; 0.5 to 0.8, moderate; and >0.8, large [37]. Data analysis was performed using the Statistical Package for Social Sciences (SPSS, version 20.0 for Windows, Chicago, IL, USA). Statistical significance was set at *p* < 0.05.

## 3. Results

**Figure 1:**
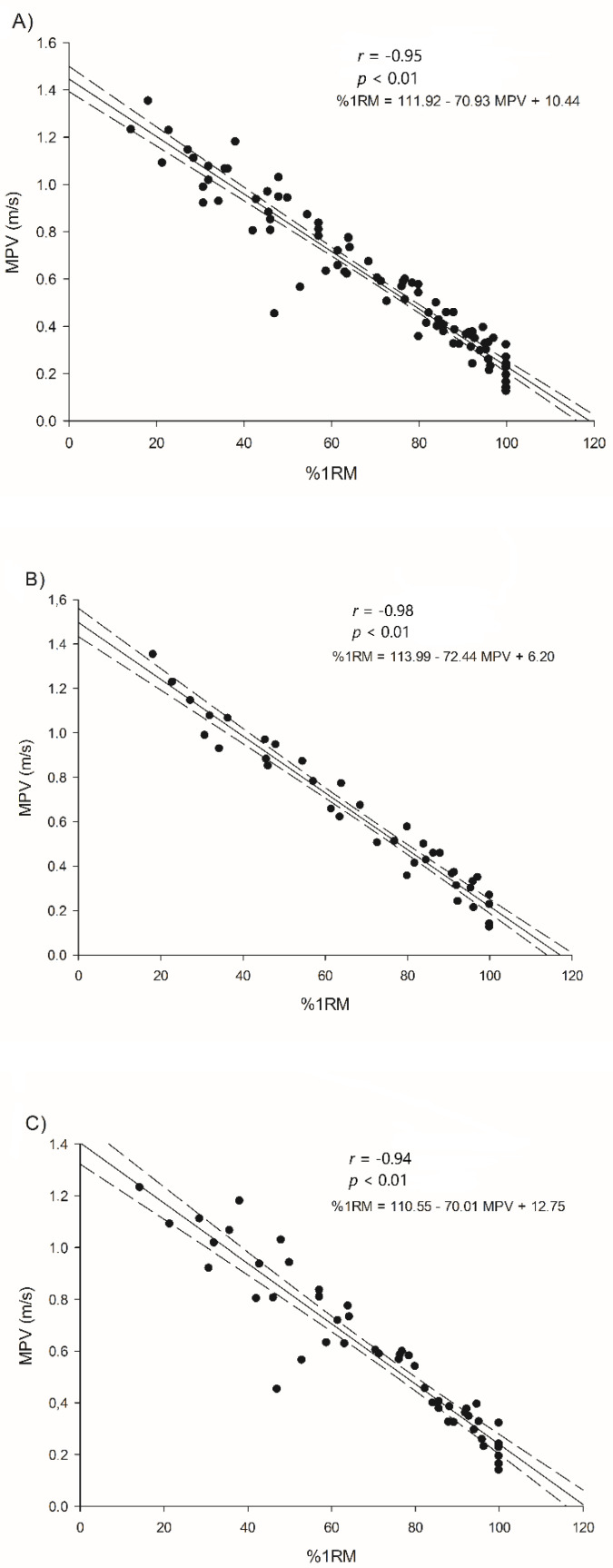
Relationships between the %1RM and the MPV of the results obtained in the pretest by all the players (**A**), the IWBF < 2.5 group (**B**), and the IWBF > 2.5 (**C**) group. r = Pearson correlation coefficient; *p* < 0.01 significant correlations between %1RM and the MPV; *p* < 0.001 significant correlations between %1RM and the MPV.

The results obtained in this study showed a high association in the pretest between the %1RM and the MPV (r = −0.96; ±0.01 CL, 0/0/100, most likely, *p* < 0.01) (Figure 1A), and between the %1RM and the V_max_ (r = −0.94; ±0.02 CL, 0/0/100, most likely, *p* < 0.01), in all players participating in the study. In the IWFB < 2.5 group, high correlations were also observed between the %1RM and the MPV (r = −0.98; ±0.01 CL, 0/0/100, most likely, *p* < 0.01) (Figure 1B) and between the %1RM and the V_max_ (r = −0.98; ±0.01 CL, 0/0/100, most likely, *p* < 0.01). Similarly, in the IWFB > 2.5 group, the results showed high associations between the %1RM and the MPV (r = −0.94; ±0.03 CL, 0/0/100, most likely, *p* < 0.05) (Figure 1C) and between the %1RM and the V_max_ (r = −0.90; ±0.05 CL, 0/0/100, most likely, *p* < 0.01).

With respect to the initial results obtained in the 1RM pretest, no significant differences were observed between the values of the IWBF < 2.5 and the IWFB > 2.5 group in any of the analyzed variables. Table 2 shows the maximum values obtained in the 1RM test, both in the pretest and in the posttest for the total of the participants, as well as for each of the groups attending to the functional classification (IWBF < 2.5 and IWBF > 2.5) and the mean difference between pretest versus posttest. After the application of a strength training program, the 1RM_Load_ of the total of participants increased significantly (*p* < 0.01, Δ% = 8.50%, ES = 0.21, small). In the same way, both the IWBF < 2.5 group (*p* < 0.01, Δ% = 6.16%, ES = 0.23, small) and the IWBF > 2.5 group (*p* < 0.01, Δ% = 10.37%, ES = 0.20, small) significantly improved their 1RM_Load_ after performing the strength training protocol.

Table 3 and Table 4 show the results obtained at submaximal intensities for the %1RM, MPV, V_max_, and TV_max_ variables obtained in the pretest and in the posttest by all the participants, as well as by each of the groups (IWBF < 2.5 and > 2.5). After the strength training protocol, significant improvements were observed in %1RM of all players in 20, 30, 40, and 60 kg. Likewise, the MPV in 30 and 40 kg and the V_max_ in 40 kg also showed significant or practical improvements (*p* < 0.05 or ES = moderate to large). With regard to the IWFB < 2.5 group, although no significant improvements were obtained in %1RM, significant improvements were observed, and improvements for practical purposes in the MPV also reached with 30, 40, 70, and 80 kg and, in the TV_max_, reached with 30, 40, and 70 kg. Conversely, in the IWBF > 2.5 group, although significant improvements were observed in the %1RM in 20, 30, and 40 kg as a consequence of the training period, no significant improvements were found in the MPV nor the TV_max_ in any of the submaximal intensities.

## 4. Discussion

The aims of this study were: (1) to investigate the associations between different mechanical variables (MPV, V_max_, and TV_max_) collected in BP exercise and distinct %1RM and (2) to analyze the effect of a strength training protocol in WB players with regard to their functional impairments (IWBF < 2.5 or > 2.5). Our data revealed a high association between the %1RM and MPV and V_max_, both in the total of participants and in each respective group of athletes (subjects with high and low functional impairment). The specific strength training program was effective for improving 1RM, for both groups of WB players (i.e., low and high functional classes). However, the training program produced positive adaptations at submaximal intensities only in the athletes with greater affectation (IWBF < 2.5).

In many sports and people with different characteristics, it has been observed a close relationship between the MPV and the %1RM [11,33,38], a feature that can facilitate the prediction of 1RM by means of the simple measurement of the MPV [16]; however, to our knowledge, this is the first study examining these associations in WB players regarding IWBF < 2.5 and IWBF > 2.5 functional classes groups. The results obtained in this study showed that, in WB players of both low functional classes (IWBF < 2.5) and high functional classes (IWBF > 2.5), there is a close correlation between the MPV and the V_max_ with the %1RM. These results are in line with those observed in the studies of Loturco et al. [38] and Iturricastillo et al. [10], where the association between the MPV and the %1RM was also high in the free execution mode in BP exercise (R^2^ = 0.95 and R^2^ = 0.94 for Loturco et al. [38] and Iturricastillo et al. [10], respectively). Bearing in mind that performing a complete and standardized test protocol to determine the 1RM in team sports requires a lot of time and has a high risk of injury [38,39], and taking into account the high association found in this study, coaches and physical trainers of WB teams could use both the MPV and the V_max_ to estimate the %1RM of their players. Currently, there are different valid and reliable tools to measure the velocity of execution of an exercise; so, to obtain these data is not complicated [11]. This aspect would facilitate the work of the practitioners, as it would allow estimating the 1RM of WB players at different times of the season in a simple way and with low risk of injury, even on a daily basis.

Various studies have shown in different sports the improvements produced by the application of resistance training programs, in athletes with or without disabilities [6,9,22,40]. However, to our knowledge, until now, there was no work in the scientific literature that analyzed the effects of strength training exclusively on high level WB players. The results of this study showed that, after the application of a strength training program, all participants improved significantly the 1RM_Load_, encompassing those with high and low functional impairments (IWBF < 2.5 and > 2.5). Strength is a relevant capacity for performance in WB, since it is associated with most relevant specific actions of the game, such as accelerations, decelerations, or changes of direction made with the wheelchair [5,6]; thus, WB coaches and physical trainers could systematically implement resistance training programs to properly develop strength-related qualities in their players.

Not only in most team sports but also in WB, the improvement of 1RM is not the only objective pursued by coaches and physical trainers. Many of the actions that occur in the game do not require maximum efforts but, rather, the ability to perform the executions at higher speeds. Therefore, it could be interesting to know if a given training program can also affect performance under “submaximal intensities”. In this context, the results of this study showed significant or practical improvements in the MPV with 30, 40, 70, and 80 kg and, in the TV_max_, with 30, 40, and 70 kg in the IWBF group < 2.5. Conversely, after performing the strength training program, the players of the IWBF > 2.5 group did not improve the MPV nor the TV_max_ in any of the submaximal loads. Consequently, the results of this study suggest that, at submaximal intensities, the training program did not produce the same adaptations in both groups, producing significant improvements in the MPV at various intensities in the group with the greatest affectation and no improvement in the remaining group. Considering that the specific strength training program was not effective for increasing the movement velocity with submaximal loads in the less impaired group (IWBF > 2.5), it would be interesting to further investigate the effects of alternative strength-power training approaches on physical (and technical) performance of WB players with lower levels of functional impairments. Practitioners involved in WB are encouraged to consider the application of different strength training schemes to achieve meaningful improvements in the strength qualities of WB players, according to their respective functional impairments.

## 5. Conclusions

The results of this study show that, due to the high association between the %1RM and MPV and V_max_, WB coaches and physical trainers could estimate the %1RM of their players in BP by measuring the MPV and the V_max_. This aspect would facilitate the process involved in carrying out a complete 1RM test protocol, also reducing the inherent risk of injury involved in maximum strength tests. On the other hand, the strength program applied herein showed to be effective for improving the maximum dynamic strength, both in players with lower or higher levels of functional impairment. However, at submaximal intensities, this training program seems to be efficient to improve MPV only in players with greater functional impairments (IWBF < 2.5). Bearing in mind that the same training program does not have the same effectiveness in both groups of WB players, it would be necessary to further investigate which type of strength training is actually effective to improve MPV in players with lower levels of functional impairment.

## Figures and Tables

**Table 1:** Bench press strength intervention program description.

	Week 1 (Set × reps)	Week 2 (Set × reps)	Week 3 (Set × reps)	Week 4 (Set × reps)	Week 5 (Set × reps)	Week 6 (Set × reps)
Day 1	3 × 6	3 × 5	3 × 5	4 × 3	4 × 3	3 × 2
Day 2	3 × 6	3 × 5	3 × 5	4 × 3	4 × 3	4 × 2
Day 3	3 × 8	3 × 6	3 × 6	3 × 4	3 × 4	3 × 3
%1RM	60%	65%	70%	75%	80%	80%

Reps = repetitions, %1RM = percentage of the 1 repetition maximum obtained in the pretest.

**Table 2:** 1RM values obtained in the pretest and in the posttest for the total of participants, as well as for each of the groups attending to the functional classification (<2.5 and >2.5).

		1RM_Load_ (kg)	1RM_MPV_ (m·s^−1^)	1RM_Vmax_ (m·s^−1^)	1RM_TVmax_ (m·s^−1^)
Total	PRE	73.50 ± 30.58	0.21 ± 0.06	0.42 ± 0.10	863.25 ± 710.79
	POST	79.75 ± 26.52 **	0.19 ± 0.09	0.40 ± 0.18	890.38 ± 930.32 *
	ES (Δ%)	0.21 (8.50)	−0.02 (−7.35)	−0.18 (−12.84)	0.04 (3.14)
IWBF < 2.5	PRE	81.25 ± 22.22	0.19± 0.07	0.42 ± 0.12	640.25 ± 772.88
	POST	86.67 ± 15.34 **	0.19 ± 0.12	0.36 ± 0.21	2102.00 ± 922.91
	ES (Δ%)	0.23 (6.16)	0.03 (0.93)	−0.48 (−14.17)	1.89 (228.31)
IWBF > 2.5	PRE	68.33 ± 36.15	0.23 ± 0.06	0.42 ± 0.09	1086.25 ± 669.99
	POST	75.42 ± 32.69 **	0.19 ± 0.07	0.43 ± 0.18	1787.68 ± 848.55
	ES (Δ%)	0.20 (10.37)	−0.04 (−12.67)	0.14 (2.86)	1.05 (64.57)

IWBF = International Wheelchair Basketball Federation classification. PRE = pretest, POST = posttest, ES = effect size, Δ% = mean difference in percentage, 1RM = 1 repetition maximum, MPV = Mean propulsive velocity, V_max_ = Maximum velocity, TVmax = Time to maximum velocity. * *p* < 0.05, ** *p* < 0.01 significant differences with the pretest.

**Table 3:** Results obtained in both the pretest and the posttest at submaximal intensities of 1RM (%1RM and MPV) by all the participants, the IWBF < 2.5 group, and the IWBF > 2.5 group.

		%1RM		MPV(m·s^−1^)		
	PRE	POST	ES (Δ%)	PRE	POST	ES (Δ%)
**TOTAL**						
20 kg (*n* = 10)	30.81 ± 10.24	27.22 ± 7.54 **	−0.35 (−11.67)	1.09 ± 0.27	1.14 ± 0.20	0.16 (4.20)
30 kg (*n* = 10)	46.21 ± 15.36	40.82 ± 11.31 **	−0.12 (−11.67)	0.93 ± 0.19	1.02 ± 0.23 **	0.43 (8.94)
40 kg (*n* = 10)	61.63 ± 20.48	54.43 ± 15.07 **	−0.35(−11.67)	0.76 ± 0.29	0.86 ± 0.23 **	0.34 (12.81)
50 kg (*n* = 8)	68.44 ± 20.20	61.68 ± 14.60	−0.33 (−9.87)	0.67 ± 0.30	0.70 ± 0.25	0.12 (5.21)
60 kg (*n* = 6)	77.51 ± 22.10	70.87 ± 16.31 *	−0.30 (−8.57)	0.55 ± 0.33	0.64 ± 0.24	0.27 (16.01)
70 kg (*n* = 3)	64.54 ± 15.02	64.12 ± 13.03	−0.03 (−0.67)	0.75 ± 0.23	0.73 ± 0.17	−0.12 (−3.59)
80 kg (*n* = 3)	73.77 ± 17.17	73.27 ± 14.89	−0.03 (−0.67)	0.59 ± 0.26	0.61 ± 0.21	0.09 (3.99)
90 kg (*n* = 2)	73.05 ± 12.40	75.00 ± 15.15	0.16 (2.67)	0.57± 0.23	0.57 ± 0.16	−0.01 (−0.18)
100 kg (*n* = 2)	81.17 ± 13.78	83.33 ± 16.84	0.16 (2.67)	0.48 ± 0.16	0.47 ± 0.20	−0.03 (−1.05)
110 kg (*n* = 1)	71.43	71.43	−(0.00)	0.59	0.61	−(3.39)
120 kg (*n* = 1)	85.72	85.72	−(0.00)	0.38	0.42	−(10.53)
130 kg (*n* = 1)	92.86	92.86	−(0.00)	0.35	0.35	−(0.00)
**IWFB < 2.5**						
20 kg (*n* = 4)	25.95 ± 6.58	23.73 ± 4.08	−0.34 (−8.56)	1.16 ± 0.16	1.15 ± 0.10	−0.08 (−1.12)
30 kg (*n* = 4)	38.93 ± 9.87	35.60 ± 6.12	−0.34 (−8.56)	0.97 ± 0.13	1.07 ± 0.15	0.81 (10.55)
40 kg (*n* = 4)	51.91 ± 13.15	47.46 ± 8.15	−0.33 (−8.56)	0.88 ± 0.19	0.98 ± 0.18*	0.53 (11.29)
50 kg (*n* = 4)	64.88 ± 16.44	59.33 ± 10.19	−0.33 (−8.56)	0.68 ± 0.28	0.73 ± 0.24	0.19 (8.44)
60 kg (*n* = 4)	77.86 ± 19.73	71.19 ± 12.23	−0.33 (−8.56)	0.56 ± 0.33	0.65 ± 0.25	0.30 (17.56)
70 kg (*n* = 2)	71.82 ± 11.57	71.17 ± 6.37	−0.05 (−0.90)	0.62 ± 0.04	0.67 ± 0.20	1.32 (7.99)
80 kg (*n* = 2)	82.08 ± 13.22	81.34 ± 7.28	−0.05 (−0.90)	0.44 ± 0.10	0.50 ± 0.15	0.70 (14.97)
90 kg (*n* = 1)	81.82	85.72	−(4.55)	0.41	0.47	−(14.64)
100 kg (*n* = 1)	90.91	95.24	−(4.76)	0.37	0.33	−(10.81)
**IWFB > 2.5**						
20 kg (*n* = 6)	34.05 ± 11.46	29.54 ± 8.72 **	−0.39 (−13.26)	1.00 ± 0.30	1.10 ± 0.25	0.35 (10.49)
30 kg (*n* = 6)	51.08 ± 17.18	44.31 ± 13.08 **	−0.39 (−13.26)	0.87 ± 0.20	0.93 ± 0.23	0.30 (6.96)
40 kg (*n* = 6)	68.11 ± 22.91	59.08 ± 17.45 **	−0.39 (−13.26)	0.68 ± 0.32	0.77 ± 0.24	0.30 (14.12)
50 kg (*n* = 4)	71.97 ± 25.46	64.03 ± 19.46	−0.31 (−11.06)	0.66 ± 0.35	0.67 ± 0.29	0.04 (1.90)
60 kg (*n* = 3)	77.06 ± 29.67	70.44 ± 23.94	−0.22 (−8.58)	0.55 ± 0.40	0.62 ± 0.28	0.19 (13.92)
70 kg (*n* = 1)	50.00	50.00	−(0.00)	1.02	0.84	−(−17.67)
80 kg (*n* = 1)	57.14	57.14	−(0.00)	0.88	0.82	−(−6.95)
90 kg (*n* = 1)	64.29	64.29	−(0.00)	0.73	0.68	−(−6.85)
100 kg (*n* = 1)	71.43	71.43	−(0.00)	0.59	0.61	−(3.39)
110 kg (*n* = 1)	78.57	78.57	−(0.00)	0.58	0.48	−(172.24)
120 kg (*n* = 1)	85.72	85.72	−(0.00)	0.38	0.42	−(10.53)
130 kg (*n* = 1)	92.86	92.86	−(0.00)	0.35	0.35	−(0.00)

PRE = pretest, POST = posttest, %1RM = percentage of the 1 repetition maximum. MPV = Mean propulsive velocity, IWBF = International Wheelchair Basketball Federation classification. Significant differences (* *p* < 0.05, ** *p* < 0.01) between pretest y posttest.

**Table 4:** Results obtained in both the pretest and the post-test at submaximal intensities of 1RM (V_max_ and TV_max_) by all the participants, the IWBF < 2.5 group, and the IWBF > 2.5 group.

	Vmax (m·s^−1^)			TVmax (m·s^−1^)		
	PRE	POST	ES (Δ%)	PRE	POST	ES (Δ%)
**TOTAL**						
20 kg (*n* = 10)	1.88 ± 0.57	2.06 ± 0.38	0.32 (9.90)	316.60 ± 67.67	317.00 ± 51.80	0.01 (0.13)
30 kg (*n* = 10)	1.56 ± 0.43	1.65 ± 0.38	0.22 (5.96)	669.30 ± 861.67	355.80 ± 91.80	−0.36 (−46.84)
40 kg (*n* = 10)	1.25 ± 0.44	1.35 ± 0.34	0.69 (7.77)	473.00 ± 187.32	461.90 ± 148.90	−0.06 (−2.35)
50 kg (*n* = 8)	1.12 ± 0.43	1.14 ± 0.36	0.06 (2.42)	622.88 ± 318.12	581.88 ± 248.55	−0.13 (−6.58)
60 kg (*n* = 6)	0.87 ± 0.47	0.99 ± 0.37	0.04 (2.19)	607.29 ± 301.22	623.72 ± 192.23	0.06 (2.71)
70 kg (*n* = 3)	1.10 ± 0.27	1.10 ± 0.30	−0.04 (−0.90)	536.00 ± 138.03	540.66 ± 106.82	0.03 (0.87)
80 kg (*n* = 3)	0.83 ± 0.30	0.90 ± 0.33	0.25 (9.35)	692.33 ± 221.72	667.00 ± 215.89	−0.11 (−3.66)
90 kg (*n* = 2)	0.82 ± 0.17	0.87 ± 0.16	0.32 (6.46)	694.00 ± 316.78	652.00 ± 179.61	−0.13 (−6.05)
100 kg (*n* = 2)	0.69 ± 0.03	0.70 ± 0.24	0.07 (0.29)	695.50 ± 679.53	847.00 ± 340.83	0.22 (21.78)
110 kg (*n* = 1)	0.72	0.86	−(19.45)	215.00	606.00	−(181.86)
120 kg (*n* = 1)	0.49	0.52	−(6.12)	214.00	300.00	−(40.19)
130 kg (*n* = 1)	0.59	0.46	−(−22.03)	179.00	315.00	−(75.98)
**IWFB < 2.5**						
20 kg (*n* = 4)	2.13 ± 0.32	2.23 ± 0.17	0.30 (4.50)	298.50 ± 56.96	305.75 ± 34.13	0.13 (2.43)
30 kg (*n* = 4)	1.72 ± 0.25	1.83 ± 0.31	0.45 (6.59)	356.00 ± 48.19	296.75 ± 21. 98	−1.23 (−16.64)
40 kg (*n* = 4)	1.43 ± 0.40	1.48 ± 0.28	0.14 (3.93)	405.25 ± 52.55	370.50 ± 57.70	−0.66 (−8.58)
50 kg (*n* = 4)	1.15 ± 0.47	1.13 ± 0.39	−0.04 (−2.00)	521.00 ± 138.45	496.00 ± 122.59	−0.18 (−4.80)
60 kg (*n* = 4)	0.87 ± 0.47	0.96 ± 0.38	0.20 (11.10)	629.25 ± 392.52	604.50 ± 196.76	−0.06 (−3.93)
70 kg (*n* = 2)	0.97 ± 0.19	0.98 ± 0.31	0.05 (1.03)	615.50 ± 13.44	572.00 ± 130.11	−3.24 (−7.06)
80 kg (*n* = 2)	0.68 ± 0.25	0.76 ± 0.31	0.31 (11.45)	813.00 ± 104.65	764.50 ± 190.21	−0.46 (−5.97)
90 kg (*n* = 1)	0.70	0.76	−(8.57)	918.00	779.00	−(−15.14)
100 kg (*n* = 1)	0.67	0.53	−(−20.90)	1176.00	1088.00	−(7.48)
**IWFB > 2.5**						
20 kg (*n* = 6)	1.71 ± 0.66	1.96 ± 0.45	0.37 (14.29)	328.67 ± 76.55	324.67 ± 62.92	−0.05 (−1.22)
30 kg (*n* = 6)	1.46 ± 0.52	1.54 ± 0.41	0.15 (5.43)	878.17 ± 1097.36	395.18 ± 101.15	−0.44 (−55.00)
40 kg (*n* = 6)	1.13 ± 0.47	1.26 ± 0.38	0.27 (11.05)	518.17 ± 235.33	522.83 ± 163.62	0.02 (0.90)
50 kg (*n* = 4)	1.08 ± 0.46	1.16 ± 0.38	0.17 (7.11)	724.75 ± 435.08	667.75 ± 330.84	−0.13 (−7.87)
60 kg (*n* = 3)	0.87 ± 0.58	1.02 ± 0.42	0.27 (17.49)	578.00 ± 197.07	649.33 ± 225.98	0.36 (12.34)
70 kg (*n* = 1)	1.38	1.33	−(3.62)	377.00	478.00	−(26.79)
80 kg (*n* = 1)	1.11	1.19	−(7.21)	451.00	472.00	−(4.66)
90 kg (*n* = 1)	0.94	0.98	−(4.26)	470.00	525.00	−(11.70)
100 kg (*n* = 1)	0.72	0.86	−(19.45)	215.00	606.00	−(181.86)
110 kg (*n* = 1)	0.82	0.69	−(−99.16)	151.00	717.00	−(374.84)
120 kg (*n* = 1)	0.49	0.52	−(6.12)	214.00	300.00	−(40.19)
130 kg (*n* = 1)	0.59	0.46	−(−22.03)	179.00	315.00	−(75.98)

PRE = pretest, POST = posttest, V_max_ = Maximum velocity, TV_max_ = Time to maximum velocity, IWBF = International Wheelchair Basketball Federation classification.

## Data Availability

Not applicable.
